# Supplementary figures and images for: Advancing aquaculture: Production of xenogenic catfish by transplanting blue catfish (Ictalurus furcatus) and channel catfish (I. punctatus) stem cells into white catfish (Ameiurus catus) triploid fry
Source: PLoS One. 2024 Jun 7;19(6):e0302687. doi: 10.1371/journal.pone.0302687 (PMC11161074; doi:10.1371/journal.pone.0302687)

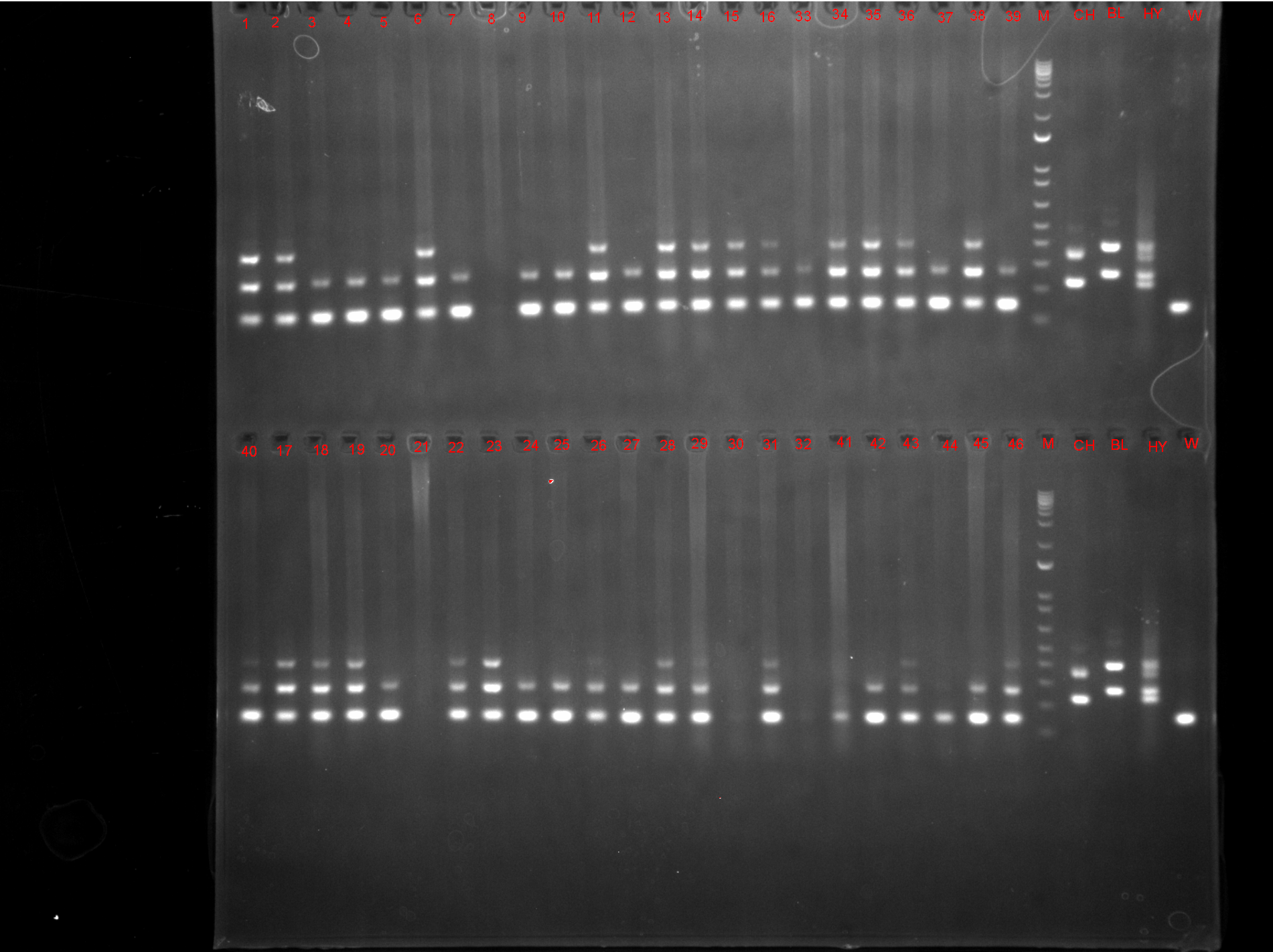

Supplement: S1 Raw image — (TIF) [file pone.0302687.s001.tif]
