# Supplementary material for: Advancing aquaculture: Production of xenogenic catfish by transplanting blue catfish (Ictalurus furcatus) and channel catfish (I. punctatus) stem cells into white catfish (Ameiurus catus) triploid fry
Source: PLoS One. 2024 Jun 7;19(6):e0302687. doi: 10.1371/journal.pone.0302687 (PMC11161074; doi:10.1371/journal.pone.0302687)
Supplement: Attachment — Submitted filename: Reviewers comments on PONE-D-23-35581-1- F.docx [file pone.0302687.s002.docx]

**Reviewer comments on the Manuscript**

As a reviewer my comments on the Manuscript narrated below for further improvement of the manuscript narrated below-

1. **Advise about the title**: Authors can think about the title alternatively appropriate like ”Advancing Aquaculture: Xenogenic Catfish Production through Blue and Channel Catfish Stem Cell Transplants in White Catfish (Ameiurus catus) Triploid Fry”
2. **The abstract** clearly outlines the objective of the study, which is to assess the effectiveness of triploid white catfish as a host for the transplantation of blue and channel catfish stem cells. It might be beneficial to include brief insights into the potential implications of these findings for commercial aquaculture and any challenges or limitations encountered during the study.
3. **In introduction** mentions that xenogenesis can mitigate challenges in hybrid catfish production, but it would be helpful to elaborate on why xenogenesis is considered a promising alternative. Highlight the specific advantages and potential improvements it offers over existing methods.

Check the consistency in terminology such as "xenogenesis" and "xenogenic." Make sure these terms are used uniformly throughout the introduction for clarity.

Clarify the rationale behind choosing triploid white catfish as the host species for xenogenesis. Explain how the unique characteristics of white catfish make it advantageous for the xenogenic process compared to channel catfish.

Addressing these points can help strengthen the introduction and provide a clearer context for the study, making it more accessible to a wider audience

4. **The methodology** is quite detailed, which is good for transparency. However, breaking it down into subsections with clear headings could enhance readability. For instance, separate sections for broodstock management, stem cell extraction, labeling, transplantation, and sample analysis would make it easier for readers to follow.

Details on the environmental conditions of the aquaculture system, such as water temperature, pH levels, and any other relevant parameters, are important for understanding the context of the study. Including information on these conditions can help readers assess the validity and generalizability of the findings.

It's mentioned that breakpoint analysis was conducted, but more details on the specific statistical tests or models used, as well as the criteria for determining breakpoints, would enhance the rigor of the methodology.

5. **In the result section**, the results are presented in a clear and organized manner. The use of figures (e.g., Fig. 3, Fig. 4) enhances the visual representation of key findings. However, it may be helpful to include concise captions for each figure to guide readers through the data.

The description of how the day of stem cell injection impacts stem cell behavior over time is well articulated. The temporal analysis provides valuable insights into the dynamics of cell and cluster areas. Ensure that the text explains the biological significance of the observed patterns, especially the peak levels and subsequent declines.

Note the consistency in the trends observed between the two types of stem cells (blue catfish and channel catfish) and how these trends compare at different time points. This helps build confidence in the reliability and reproducibility of the experimental outcomes.

Continue to interpret the data in the context of the study's objectives and the broader implications for xenogenic catfish production. Discuss how the observed trends align with the goals of the experiment and any potential applications or challenges identified.

Consider including a brief section or concluding remarks discussing potential implications of the results for future research or practical applications in aquaculture.

6. **The discussion** is clear and well-organized, addressing the key findings step by step. However, consider providing a brief overview or roadmap at the beginning of the discussion to guide readers through the main points and structure of the discussion.

How was the specific age range of 4.0 to 5.0 days post-hatch (DPH) identified as optimal for stem cell transplantation into white catfish hosts, and what factors were considered in making this determination?

The discussion acknowledges technical challenges during the early stages of stem cell transplantation. Can you elaborate on specific challenges faced during injection into fry and whether these challenges are inherent to the procedure or related to the life stage of the fish?

The discussion suggests recirculating aquaculture systems (RAS) as a potential alternative. Could you delve deeper into how RAS could overcome the identified issues of harvestability and disease resistance in white catfish, and are there any specific challenges associated with this alternative?

7. Is there any ethical consideration permission required for this study ? or not applicable in this case?
