# Supplementary material for: Advancing aquaculture: Production of xenogenic catfish by transplanting blue catfish (Ictalurus furcatus) and channel catfish (I. punctatus) stem cells into white catfish (Ameiurus catus) triploid fry
Source: PLoS One. 2024 Jun 7;19(6):e0302687. doi: 10.1371/journal.pone.0302687 (PMC11161074; doi:10.1371/journal.pone.0302687)
Supplement: Attachment — Submitted filename: Review PlosOne_Simora.docx [file pone.0302687.s003.docx]

Title: **Production of xenogenic catfish by transplanting blue catfish (*Ictalurus furcatus*) and channel catfish (*I. punctatus*) stem cells into white catfish (*Ameiurus catus*) triploid fry**

General Comments to the Authors:

The paper assessed the effectiveness of white catfish as host species to transplant blue catfish and channel catfish stem cells through xenogenesis. The authors can improve the manuscript by improving the labels in the figures, making sure that the values described in the Results section coincide with those in the figures. The Discussion can be improved by including pertinent information such as the basis/criteria for choosing the host fish for xenogenesis, compare the present data on survival of xenogens and efficiency of xenogenesis with previous data with blue and channel catfish (if any), and explain the reasons why the transplantation efficiency was greatest between 4 and 5 DPH.

Specific Comments:

| **Line** | **Comments** |
| --- | --- |
| 21-22 | Please make clear in this statement if you are referring to the white catfish as having short maturation time and small body size. |
| 49-50 | Insert hybrid “catfish” industry. |
| 89 | Correct “xenogenic” spelling. |
| 225-228 | Were the results for 4 and 4.5 as well as the 5.5 DPH shown in Fig.4? What was the reason for getting the survival at 4.5 and 5.5 DPH, and how was it different from 4 and 5 DPH, respectively? |
| 242-243 | The values for 8.6 and 9.2 DPH is not reflected in the Fig. B. It is only the values for 8 and 9 DPH. I think the labelling for the x-axis in Fig. 3,4, 5 and 6 is quite confusing. Do you refer to “days of injection” as “days post hatch”? |
| 243-244 | What is the difference between cell area and cluster area? |
| 276-278 | Do you have the basis/reason for these criteria? Include in the discussion. Were there published results on doing xenogenesis on blue and channel catfish? How was the survival of xenogens on these species? Compare it with your present data. |
| 282-285 | Are these statements necessary, since you did not compare PKH26 with other dyes? Please consider revising or just delete. |
| 289-291 | Should explain the differences between cell and cluster areas and what should be the “ideal cell and cluster area” look like. |
| 291-293 | Include in the Discussion the reasons why between 4 and 5 DPH the transplantation efficiency was greatest. |
| 325-326 | Did you also observe low survival of white catfish transplanted with xenogens in the present study? Please include in the discussion. |
| 398-400 | Some missing label information for CH, BL, HY in Fig. 7. |
